# Interventions for posttraumatic stress disorder in psychiatric practice across Europe: a trainees’ perspective

**DOI:** 10.3402/ejpt.v6.27818

**Published:** 2015-09-07

**Authors:** Katja Koelkebeck, Olivier Andlauer, Nikolina Jovanovic, Domenico Giacco

**Affiliations:** 1 Department of Psychiatry and Psychotherapy, University of Muenster, Muenster, Germany; 2 Newham Centre for Mental Health, East London NHS Foundation Trust, London, United Kingdom; 3 Department of Psychiatry, University Hospital Center Zagreb, Zagreb, Croatia; 4 Unit for Social and Community Psychiatry, Barts and the London School of Medicine and Dentistry, Queen Mary University of London, London, United Kingdom

**Keywords:** educational status, healthcare surveys, psychotherapy, PTSD

## Abstract

**Background:**

With an annual prevalence of 0.9–2.6%, posttraumatic stress disorder (PTSD) is very common in clinical practice across Europe. Despite the fact that evidence-based interventions have been developed, there is no evidence on their implementation in clinical practice and in national psychiatric training programmes.

**Objective and method:**

The Early Career Psychiatrists Committee of the European Psychiatric Association conducted a survey in 23 European countries to explore implementation of evidence-based interventions for PTSD and training options.

**Results:**

The findings indicate that pharmacotherapy was available in the majority of the participating countries (*n*=19, 82.8%). However, psychological interventions were much less widespread. For example, psychoeducation was widely available in 52% of the countries (*n*=12), cognitive-behavioural therapy in 26.2% (*n*=6), and specific trauma-focused techniques were rarely available. Training on PTSD was part of the official training in 13 countries (56.5%), predominantly in the form of theoretical seminars.

**Conclusions:**

Overall, this survey indicates that the treatment for PTSD is largely focused on pharmacotherapy, with psychological evidence-based interventions poorly available, especially outside specialized centres. Poor implementation is linked to the lack of official training in evidence-based interventions for psychiatric trainees across Europe.

Posttraumatic stress disorder (PTSD) is frequent in the general population. In recent European surveys, lifetime prevalence rates of 1.9% (Alonso et al., 2004) and 12-month prevalence rates of 0.9–2.6% were identified (Darves-Bornoz et al., 2008). Evidence-based interventions to reduce psychological distress in patients with PTSD include pharmacotherapy (Ravindran & Stein, 2010), psychoeducation (Asukai, Tsuruta, & Saito, 2011; Oflaz, Hatipoglu, & Aydin, 2008) and cognitive-behavioural therapy (CBT) (Bryant et al., 2011) as well as trauma-focused techniques such as eye movement desensitization and reprocessing, stress management and group trauma-focused CBT (Bisson & Andrew, 2007; Roberts, Kitchiner, Kenardy, & Bisson, 2010). Guidelines and specific interventions have been designed for the treatment of PTSD (Bisson, 2013; Vymetal et al., 2011), but it remains unclear whether evidence-based treatments are implemented in countries across Europe and if training options are available.

The significant prevalence of PTSD in the population implies that trainees and early career psychiatrists (ECP: defined as aged under 40 and/or within 5 years of finishing specialty training) often encounter PTSD patients in their clinical practice. Psychologists and allied professionals in the USA reported that their training in the treatment of PTSD was not sufficient to meet the needs in clinical practice (Cook, Rehman, Bufka, Dinnen, & Courtois, 2011; Courtois, 2001; Courtois & Gold, 2009), which is why measures have been taken to spread evidence-based therapies (Karlin et al., 2010). This might also apply to psychiatrists, specifically as psychiatric training assumingly focuses less on psychotherapeutic interventions (Yager & Kay, 2003). In this study, we aimed to explore the implementation of evidence-based treatments for PTSD in clinical practice and training curricula across Europe.

## Method

To explore the availability of evidence-based interventions for PTSD and their presence in training curricula, we carried out a survey among national psychiatric trainee representatives in 23 European countries. Our aim was to determine: (1) how often they had contact with PTSD patients in their clinical practice; (2) whether evidence-based interventions were available in the majority of centres in their country; and (3) whether training options in PTSD treatment were included in training curricula and if so, in what form.

To this end, a questionnaire was developed by members of the ECP committee of the European Psychiatric Association (EPA-ECPC) and members of its research task force. The questionnaire was developed during three online sessions attended by psychiatric trainees from seven European countries. The questionnaire was in English. It consisted of two large categories of interest: the availability of evidence-based interventions (eight questions) and the availability of training in evidence-based treatment options in each country (seven questions, Table 1). In case evidence-based interventions for PTSD were not available, we asked participants to provide information on possible reasons. To assess the exposure of respondents to patients with PTSD, we posed a question regarding the frequency of contact to patients. Demographic data of participants, work qualification, as well as the availability of and the impact of national guidelines on evidence-based treatment options for PTSD, were additionally assessed. The questionnaire consisted of yes/no answers and five-point scales regarding availability of treatments or training. The circulation of the questionnaire was approved by the EPA Board, and the study was conducted between July and October 2012. Presidents of national psychiatric trainee or ECP associations recognized by the EPA in 35 European countries (including ECP sections of major psychiatric associations in the respective countries) were contacted via email and invited to participate. Presidents were asked to act as “national experts” or identify a psychiatric trainee or ECP expert among the officers of their association. They were encouraged to consult all available sources to obtain the most accurate data and were asked to participate only if they had significant clinical and/or research experience in this field. This methodology was adopted to provide collective input of their trainees’ association and official training curriculum rather than the experience or perspective of any individual officer or member.

**Table 1:** Availability of evidence-based interventions for PTSD and training options in participating countries

	Evidence-based intervention					
	
Country	Psychopharmacology	Psychoeducation	CBT	EMDR	Stress reduction	Group therapy
Albania	All	Specialized	Specialized	*–*	*–*	*–*
Azerbaijan	Majority	Specialized	Rarely	Rarely	Rarely	Rarely
Belarus	All	Majority	Few	Rarely	Few	Rarely
Bosnia and Herzegovina	All	Specialized	Specialized	Specialized	Specialized	Specialized
Croatia	All	Majority	Few	Specialized	Specialized	Specialized
Czech Republic	All	Majority	Few	Specialized	Few	Specialized
Finland	All	Majority	Majority	Few	All	Few
France	Majority	Few	Majority	Specialized	Few	Specialized
Germany	All	Majority	Specialized	Specialized	All	Few
Greece	Few	Specialized	Specialized	Rarely	Few	Specialized
Latvia	All	Few	Rarely	Rarely	Rarely	Rarely
Lithuania	All	Few	Few	Rarely	Rarely	Rarely
Malta	All	All	Rarely	Rarely	Majority	Rarely
The Netherlands	All	All	All	Majority	All	Few
Poland	Few	Majority	Few	Rarely	Few	Rarely
Portugal	All	Few	Majority	Rarely	Few	Few
Romania	Majority	Majority	Few	Rarely	Few	Rarely
Russia	All	Specialized	Specialized	Rarely	Specialized	Specialized
Serbia	All	All	Majority	Few	All	Specialized
Slovenia	All	All	Few	Rarely	Few	Specialized
Switzerland	All	All	All	Few	All	Few
Turkey	Few	Few	Few	Rarely	Few	Specialized
Ukraine	All	Rarely	Few	Rarely	Few	Few

All=in nearly all centres; Majority=in the majority of centres; Few=in a few centres; Specialized=only in specialized centres; Rarely=very rarely available; –=no treatment/training available; CBT, cognitive-behavioural therapy; EMDR, eye movement desensitization and reprocessing.

### Data analysis

Data on socio-demographic and work-related characteristics, clinical exposure to PTSD patients, availability of evidence-based interventions, and training options across the countries were analysed by descriptive statistics. Analyses were performed with SPSS V19.0 (SPSS, Inc., Chicago, IL, USA).

## Results

Experts from 23 out of 35 countries completed the survey (Table 1). Of these, 10 were presidents of the respective national ECP organizations. Participants were predominantly female (*n*=15, 65.2%) with a mean age of 32.3 years (*SD*=3.7). They were either psychiatric trainees (*n*=11, 47.8%) or ECP (*n*=12, 52.2%). The majority of respondents (*n*=17, 73.9%) reported seeing at least one patient with PTSD in the previous month, whereas eight participants (34.8%) reported seeing one in the previous week. National guidelines for treatment were available in 11 countries (47.8%). Their impact on practice was considered as “high” in only four countries (Croatia, Netherlands, Romania, and Switzerland). Availability of evidence-based interventions for PTSD treatment is summarized in Table 1. Training was provided in the majority of training centres in the form of theoretical seminars (*n*=10, 43.5%), discussion of clinical cases (*n*=9, 39.1%), individual supervision (*n*=7, 30.4%), group supervision (*n*=3, 13.0%) and continuing medical education (CME) courses (*n*=5, 21.7%). The reasons for a poor implementation of evidence-based practice reported by experts (more than one answer was possible) were: lack of funding (*n*=8, 34.7%), lack of expertise in the country (*n*=8, 34.7%), poor recognition and identification of trauma-related disorders by psychiatrists (*n*=3, 13.1%) and problems with organization of mental health care (*n*=3, 31.1%). Four experts did not report problems in implementation in their countries (Finland, Germany, Portugal and Romania).

## Discussion

The data gathered by national experts represent a snapshot of the current provision of evidence-based treatments for PTSD and of their presence in national training curricula in European countries. Our findings emphasize the need for strategies to increase the availability of evidence-based interventions for PTSD in European countries. Pharmacological interventions were the most frequently available type of treatment for PTSD across European countries. This is not surprising given that psychopharmacology is an integral part of psychiatric training. Although psychoeducation was widely available in more than 50% of the countries, CBT and other specific interventions were most often provided only in specialized centres. A wider availability of training options for evidence-based treatment outside specialized centres was given mostly in a few Western European countries (Finland, Germany, Netherlands and Malta) with the exception of Serbia. A lack of funding and lack of expertise in the countries were the most common reasons given for lack of implementation of evidence-based practices. Even when training was available in national training curricula, it was mainly based on theoretical seminars. In only 30% of the countries, CME initiatives on PTSD treatment were offered. These findings echo previous studies, which emphasize major differences in psychiatric training curricula across Europe (Lotz-Rambaldi, Schafer, Ten Doesschate, & Hohagen, 2008) and clearly demand for improvement. Theoretical seminars are not enough to facilitate the development of expertise in clinical practice. Training curricula should involve not only formal lectures but also interactive workshops as well as procedures to validate and monitor practice (Toot, Orrell, Rymaszewska, & Ihl, 2012). There is evidence that training can improve the detection of trauma-related disorders (Frueh et al., 2002) and might help psychiatric trainees delivering first-aid CBT (Hamblen, Norris, Gibson, & Lee, 2010). Possible steps towards the implementation of evidence-based treatments for PTSD may be helped by initiatives on a European-wide level. A number of initiatives are already offered, for example, the Certificate in Psychotrauma and the European Network for Traumatic Stress of the European Society for Traumatic Stress Studies (Bisson, 2013) or the European Guideline for Target Group-Oriented Psychosocial Aftercare (Vymetal et al., 2011). “Summer schools” as organized by the EPA (Riese, Pantovic, Fiorillo, Tasman, & Sartorius, in press) might act as models for cross-European training initiatives. Potential language barriers could be overcome if similar approaches were implemented on national levels. Internet-based trainings and training of mentors are other strategies proposed (Ruzek & Rosen, 2009). The provision of funding of such initiatives should take into account that qualified treatment of PTSD can reduce disability through chronic disease (Ehlers, Clark, Hackmann, McManus, & Fennell, 2005).

Limitations of our study include an only satisfactory response rate (23 out of 35) with a high prevalence of responses from eastern European countries (14 out of 23). Moreover, as one expert per country answered the survey and we had to rely on the expertise of the respondents, results may have been biased. Yet, surveys of nationally appointed experts are commonly used to obtain representative data (Kuzman et al., 2012; Lotz-Rambaldi et al., 2008) and participants were well informed on the situations in their country. Given the fact that only very limited data on training options in the respective countries were available, it is advisable that societies on psychotraumatology conduct larger scale investigations of training options across Europe.

Strategies to increase the availability of evidence-based interventions for PTSD across European countries are needed. Ensuring that the highest standard of training on PTSD treatment is part of national psychiatric curricula is an important component of these strategies. Lack of trained professionals may reduce the likelihood of PTSD detection and limit the provision of evidence-based treatments to specialized centres.

## Supplementary Material

Interventions for posttraumatic stress disorder in psychiatric practice across Europe: a trainees’ perspective

Interventions for posttraumatic stress disorder in psychiatric practice across Europe: a trainees’ perspective

Interventions for posttraumatic stress disorder in psychiatric practice across Europe: a trainees’ perspective

Interventions for posttraumatic stress disorder in psychiatric practice across Europe: a trainees’ perspective
