# Supplementary material for: Interventions for posttraumatic stress disorder in psychiatric practice across Europe: a trainees’ perspective
Source: Eur J Psychotraumatol. 2015 Sep 7;6:10.3402/ejpt.v6.27818. doi: 10.3402/ejpt.v6.27818 (PMC4563100; doi:10.3402/ejpt.v6.27818)
Supplement: Interventions for posttraumatic stress disorder in psychiatric practice across Europe: a trainees’ perspective [file EJPT-6-27818-s001.pdf]

Metody nterwencji nakierowanych na leczenie zaburzenia po stresie traumatycznym: praktyki pochodzące z różnych krajów europejskich.

Katja Koelkebeck, Olivier Andlauer, Nikolina Jovanovic, Domenico Giacco

Wprowadzenie: Roczny odsetek występowania PTSD w róznych krajach europejskich wynosi śrdnio 0.9-2.6%. Mimo że wypracowano wiele opartych na badanich naukowych metod leczenia PTSD, badania pokazują, że metody te rzadko są stosowane w praktyce klinicznej.
Cel i metoda: Przeprowadzono ankietę w 23 krajach europejskich w celu oszacowania skali rzeczywistego wykorzystania w praktyce klinicznej opartych na badanich naukowych metod interwencji nakierowanych na leczenie PTSD.
Wyniki: Dane wskazują, że najbardziej rozpowszechnioną i wykorzystywaną formą leczenia PTSD jest farmakoterpia (n = 19, 82.8%). Interwencje psychologiczne wykorzystywane są znacznie rzadziej: psychoedukacja (n = 12, 52%), terapia poznawczo-behawioralna (n = 6, 26.2%). Natomiast specjalistyczne treningi w kierunku radzenia sobie z PTSD odnotowano w 13 krajach (56.5%).
Konkluzje: Dane wskauzują, że najbardziej rozpowszechnioną metodą interwencji nakierowaną na leczenie PTSD jest farmakoterapia, podczas gdy oparte na danych naukowych metody interwencji psychologicznych są stosowane znacznie rzadziej.

Słowa kluczowe: status edukacyjny; ankieta zdrowotna; psychoterapia; PTSD.

Name of translator: Marcin Rzeszutek, University of Finance and Management in Warsaw, Poland

Citation: European Journal of Psychotraumatology 2015, **6**: 27818 - http://dx.doi.org/10.3402/ejpt.v6.27818
