# Supplementary material for: Interventions for posttraumatic stress disorder in psychiatric practice across Europe: a trainees’ perspective
Source: Eur J Psychotraumatol. 2015 Sep 7;6:10.3402/ejpt.v6.27818. doi: 10.3402/ejpt.v6.27818 (PMC4563100; doi:10.3402/ejpt.v6.27818)
Supplement: Interventions for posttraumatic stress disorder in psychiatric practice across Europe: a trainees’ perspective [file EJPT-6-27818-s002.pdf]

Avrupa boyunca psikiyatri pratiğindeki travma sonrası stresteki müdahaleler: Bir stajyerin bakış açısı

Katja Koelkebeck, Olivier Andlauer, Nikolina Jovanovic, Domenico Giacco

Abstract
Arkaplan: Yıllık 0.9-2.6% yaygınlık ile, travma sonrası stres bozukluğu (TSSB) Avrupa boyunca klini pratiğinde oldukça yaygın. Kanıt temelli müdahaleler geliştirilmesine rağmen , klinik kullanımına ve ulusal psikiyatrik eğitim programlarında uygulanmasında bir kanıt yok. Amaç ve Yöntem: Avrupa Psikiyatri Derneği Erken Kariyer Psikiyatri Komitesi (The Early Career Psychiatrist Committee of the European Psychiatric Association) TSSB ve eğitim seçenekleri için kanıt temelli müdahalelerin uygulanmasını araştırmak için 23 Avrupa ülkesinde bir anket yürütmüştür. Sonuçlar: Bulgular, katılımcı ülkelerin çoğunluğunda farmakoterapinin ulaşılabilir olduğunu göstermiştir (n= 19, 82,8 %). Buna rağmen, psikolojik müdahaleler daha az yaygındır. Örneğin, psikoeğitim ülkelerin (n=12) % 52'sinde geniş bir şekilde yaygındır, bilişsel-davranışçı terapi %26.2 (n=6); spesifik travma-odaklı tenikler oldukça nadir olarak bulunmaktadırlar. TSSB üzerine eğitim, ağırlıklı olarak teorik seminerler şeklinde 13 ülkede (% 56.5) resmi eğitimin parçasıdır. Tartışma: Sonuç olarak, bu anket TSSB için olan tedavinin büyük çoğunlukla farmakoterapi üzerine odaklandığını gösterirken, psikolojik kanıta dayalı müdahalelerin, özellikle dışa odaklı uzmanlaşmış merkezlerde, oldukça az olduğunu göstermiştir. Az uygulanmanın Avrupa boyunca psikiyatri stajyerleri için olan kanıta dayalı müdahalelerdeki resmi eğitimin eksik olması ile ilgilidir.

Anahtar Kelimeler:
Eğitim Statüsü; Sağlık hizmeti anketleri; psikoterapi; TSSB

Name of translator: Seray Akça

Citation: European Journal of Psychotraumatology 2015, **6**: 27818 - http://dx.doi.org/10.3402/ejpt.v6.27818
