# Supplementary material for: Interventions for posttraumatic stress disorder in psychiatric practice across Europe: a trainees’ perspective
Source: Eur J Psychotraumatol. 2015 Sep 7;6:10.3402/ejpt.v6.27818. doi: 10.3402/ejpt.v6.27818 (PMC4563100; doi:10.3402/ejpt.v6.27818)
Supplement: Interventions for posttraumatic stress disorder in psychiatric practice across Europe: a trainees’ perspective [file EJPT-6-27818-s003.pdf]

Interventionen bei Posttraumatischen Belastungsstörungen in der psychiatrischen Praxis in Europa: die Persepektive Auszubildender

Katja Koelkebeck, Olivier Andlauer, Nikolina Jovanovic, Domenico Giacco

Hintergrund: Mit einer Jahresprävalenz von 0.9-2.6% ist das Auftreten einer posttraumatischen Belastungsstörung (PTBS) in der klinischen Praxis in Europa sehr häufig. Obwohl evidenzbasierte Interventionen entwickelt wurden, gibt es keine Belege für deren Implementierung in der klinischen Praxis und in nationalen psychiatrischen Ausbildungsprogrammen.
Ziel und Methode: Das "Early Carreer Psychiatrist Committee" der "European Psychiatric Association" führte in 23 europäischen Ländern eine Studie über die Implementierung von evidenzbasierten Interventionen bei PTBS und deren Ausbildungsmöglichkeiten durch.
Ergebnisse: Die Ergebnisse weisen darauf hin, dass pharmakologische Therapien in der Mehrheit der teilnehmenden Länder verfügbar waren (n = 19, 82.8%). Hingegen waren psychologische Interventionen deutlich weniger weit verbreitet. So war Psychoedukation beispielsweise in 52% der Länder weitgehend verfügbar, kognitive Verhaltenstherapie in 26.2% (n = 6) und spezifische Techniken der Traumabehandlung waren kaum verfügbar. Schulungen zu PTBS waren in 13 Ländern Teil der offiziellen Ausbildung (56.5%), mehrheitlich in Form von theoretischen Seminaren.
Schlussfolgerungen: Insgesamt zeigte diese Studie, dass sich die Behandlung von PTBS weitgehend auf pharmakologische Therapien konzentriert, während evidenzbasierte psychologische Interventionen vor allem außerhalb von spezialisierten Einrichtungen sehr unzureichend verfügbar sind. Die geringe Implementierung kann auf den Mangel an offiziellen Schulungen zu evidenzbasierten Intervention in psychiatrischen Ausbildungen in Europa zurückgeführt werden.

Keywords: Bildungsstand; Gesundheitserhebungen; Psychotherapie; PTBS;

Name of translator: Theresa Leitner, Brigitte Lueger-Schuster

Citation: European Journal of Psychotraumatology 2015, **6**: 27818 - http://dx.doi.org/10.3402/ejpt.v6.27818
