# Supplementary material for: Interventions for posttraumatic stress disorder in psychiatric practice across Europe: a trainees’ perspective
Source: Eur J Psychotraumatol. 2015 Sep 7;6:10.3402/ejpt.v6.27818. doi: 10.3402/ejpt.v6.27818 (PMC4563100; doi:10.3402/ejpt.v6.27818)
Supplement: Interventions for posttraumatic stress disorder in psychiatric practice across Europe: a trainees’ perspective [file EJPT-6-27818-s004.pdf]

Final but unedited version

Citation: European Journal of Psychotraumatology 2015, **6**: 27818 - http://dx.doi.org/10.3402/ejpt.v6.27818

Therapie von posttraumatischen Belastungsstörungen in Europa: Eine Perspektive psychiatrischer Assistenzärzte

Katja Koelkebeck[1], Olivier Andlauer[2], Nikolina Jovanovic[3], Domenico Giacco[4]

[1]University of Muenster, Department of Psychiatry and Psychotherapy, Muenster, Germany

[2]Newham Centre for Mental Health, East London NHS Foundation Trust, London, United Kingdom

[3]Department of Psychiatry, University Hospital Center Zagreb, Zagreb, Croatia

[4]Unit for Social and Community Psychiatry, Barts and the London School of Medicine and Dentistry, Queen Mary University of London, London, United Kingdom

E-Mail-Adressen: giaccod@gmail.com; koelkebeck@uni-muenster.de; nikolina.jovanovic@gmail.com;olivier.andlauer@gmail.com

Korrespondenzadresse: Katja Koelkebeck, Klinik für Psychiatrie und Psychotherapie, Universitätsklinikum Münster, Albert-Schweitzer-Campus 1, Gebäude A 9, 48149 Münster, Deutschland, Tel: 0049-251-8456601, Fax: 0049-251-8356612; E-Mail: koelkebeck@uni-muenster.de

Abstract

Hintergrund: Mit einer jährlichen Prävalenz von 0,9-2,6% ist die posttraumatische Belastungsstörung (PTBS) eine häufige Diagnose in der klinischen Praxis in Europa. Trotz der Tatsache, dass evidenzbasierte Therapien verfügbar sind, ist die Frage nach deren Umsetzung in der klinischen Praxis und in nationalen Ausbildungsprogrammen für Psychiater ungeklärt. Ziele und Methoden: Durch das Early Career Psychiatrist Committee der European Psychiatric Association wurde in 23 europäischen Ländern eine Umfrage durchgeführt, deren Ziel es war, die Implementierung von evidenzbasierten Therapien bei PTBS und auch Ausbildungsmöglichkeiten zu explorieren. Ergebnisse: Die Ergebnisse zeigen, dass eine Pharmakotherapie die Therapieoption ist, die in allen teilnehmenden Ländern verfügbar war (n = 19; 82,8%). Psychotherapeutische Behandlungen waren jedoch deutlich weniger verbreitet. Psychoedukation war z. B. in 52% der Länder verfügbar (n = 12), kognitiv-behaviorale Therapie in 26,2% (n = 6). Spezifische, traumafokussierte Therapien waren nur selten verfügbar. Die PTBS war nur in 13 Ländern Gegenstand der offiziellen Ausbildung (56,5%), vorranging in Form theoretischer Seminare. Schlussfolgerungen: Die hier durchgeführte Umfrage ergibt zusammenfassend, dass die Behandlung der PTBS sich vornehmlich auf Pharmakotherapie fokussiert, während evidenzbasierte psychotherapeutische Behandlungen außerhalb spezialisierter Einrichtungen nur unzulänglich verfügbar sind. Eine unzureichende Implementierung evidenzbasierter Therapien steht in direktem Zusammenhang mit reduzierten offiziellen Ausbildungsmöglichkeiten für psychiatrische Assistenzärzte in Europa.

Schlüsselwörter:

Ausbildungsstatus; Gesundheitsstudie; Psychotherapie; PTBS

Hintergrund

Posttraumatische Belastungsstörungen (PTBS) sind in der Allgemeinbevölkerung häufig. Nach aktuellen europäischen Erhebungen beträgt die Lebenszeitprävalenz 1,9% (Alonso et al., 2004) und eine 12-Monatsprävalenz von 0,9-2,6% wurde identifiziert (Darves-Bornoz et al., 2008). Evidenzbasierte Therapien zur Reduktion von psychischem Stress bei Patienten mit PTBS beinhalten Pharmakotherapie (Ravindran & Stein, 2010), Psychoedukation (Asukai, Tsuruta, & Saito, 2011, Oflaz, Hatipoglu, & Aydin, 2008), kognitiv-behaviorale Therapie (CBT) (Bryant et al., 2011) und traumafokussierte Techniken wie Eye Movement Desensitization and Reprocessing (EMDR), Stress Management und traumafokussierte CBT Gruppentherapie (Bisson & Andrew, 2007, Roberts, Kitchiner, Kenardy, & Bisson, 2010). Für die Behandlung von PTBS wurden Richtlinien und spezifische Therapien entwickelt (Bisson, 2013, Vymetal et al., 2011), es bleibt jedoch unklar, ob evidenzbasierte Behandlungen in den europäischen Ländern implementiert und ob Ausbildungsmöglichkeiten vorhanden sind.

Die hohe Prävalenz der PTBS in der Allgemeinbevölkerung legt nahe, dass psychiatrische Assistenzärzte und Psychiater in den frühen Stadien ihrer Karriere (Early Career Psychiatrists (ECP): definiert als unter 40 Jahre und vor dem oder bis zu fünf Jahre nach dem Facharzt) in ihrer klinischen Praxis oft auf Patienten mit einer PTBS treffen. Psychologen und fachverwandte Berufsgruppen in den USA berichten, dass die Ausbildung in der Behandlung der PTBS den Erfordernissen der klinischen Praxis nicht nachkommt (Cook, Rehman, Bufka, Dinnen, & Courtois, 2011, Courtois, 2001, Courtois & Gold, 2009), weswegen dort Maßnahmen ergriffen wurden, um konsequent evidenzbasierte Therapien zu verbreiten (Karlin et al., 2010). Dies trifft vermutlich auch auf Psychiater zu, besonders, da in der psychiatrischen Ausbildung der Fokus weniger stark auf psychotherapeutische Therapien gelegt wird (Yager & Kay, 2003). Mit dieser Studie wollen wir die Implementierung

evidenzbasierter Therapien bei PTBS in der klinischen Praxis und in Ausbildungscurricula in Europa explorieren.

Methoden

Um die Verfügbarkeit von evidenzbasierten Therapien bei PTBS und deren Präsenz in Ausbildungscurricula zu explorieren, führten wir eine Befragung unter Repräsentanten nationaler psychiatrischer Assistenzärzte in 23 europäischen Ländern durch. Unsere Ziele waren zu determinieren: a) wie oft diese in ihrer klinischen Praxis Kontakt mit PTBS Patienten haben; b) ob evidenzbasierte Therapien in der Mehrzahl der Ausbildungszentren des entsprechenden Landes verfügbar sind; c) ob Ausbildungsmöglichkeiten für die Behandlung von PTBS ein Bestandteil der Ausbildungscurricula sind, und wenn ja, in welcher Form.

Es wurde daher ein Fragebogen von Mitgliedern des Early Career Psychiatrist Committee der European Psychiatric Association (EPA-ECPC) und dessen Task Force Research entworfen. Der englischsprachige Fragebogen wurde von psychiatrischen Assistenzärzten aus sieben Ländern während computergestützter Online-Treffen entwickelt. Er besteht aus zwei übergeordneten Interessenskategorien: die Verfügbarkeit evidenzbasierter Therapien (acht Fragen) und die Verfügbarkeit von Ausbildungsmöglichkeiten evidenzbasierter Therapien in den entsprechenden Ländern (sieben Fragen, siehe **Tabelle 1**). Für den Fall, dass evidenzbasierte Therapien bei PTBS nicht verfügbar waren, sollten die Teilnehmer Angaben zu den möglichen Ursachen machen. Um die Exposition der Teilnehmer gegenüber Patienten mit einer PTBS zu überprüfen, wurde eine Frage zur Frequenz von Kontakten zu Patienten eingefügt. Demographische Daten zu den Teilnehmern, zu deren beruflichen Qualifikation sowie zu der Verfügbarkeit und dem Einfluss nationaler Richtlinien evidenzbasierter Therapien bei PTBS wurden ebenfalls erhoben. Der Fragebogen bestand aus Ja/Nein Antworten sowie einer 5-Punkte Skala bezüglich der Verfügbarkeit von Therapien und Trainingsmöglichkeiten. Der Einsatz des Fragebogens wurde durch den EPA Vorstand autorisiert, die Umfrage wurde zwischen Juli und Oktober 2012 durchgeführt. Die Vorsitzenden der durch die EPA anerkannten nationalen Assistenten- oder ECP

Organisationen in 35 europäischen Ländern (einschließlich der ECP Sektionen der großen psychiatrischen Fachgesellschaften in den entsprechenden Ländern) wurden über E-Mail kontaktiert und zur Teilnahme eingeladen. Die Vorsitzenden sollten als "nationale Experten" fungieren oder einen Assistenzarzt oder ECP unter den Vorstandsmitgliedern ihrer Organisation identifizieren. Sie wurden aufgefordert, alle zur Verfügung stehenden Quellen heranzuziehen, um möglichst genaue Daten zu liefern, und wurden gebeten, nur teilzunehmen, wenn sie signifikante klinische oder Forschungserfahrung in diesem Bereich vorweisen konnten. Dieses Vorgehen wurde gewählt, um eine kollektive Rückmeldung von Assistenzarztorganisationen und über offizielle Ausbildungscurricula zu erhalten statt einer subjektiven Erfahrung und Perspektive einzelner Vorstandsmitglieder.

Datenanalyse

Soziodemographische und arbeitsbezogene Charakteristika, klinische Exposition gegenüber PTBS Patienten, Verfügbarkeit evidenzbasierter Therapien und Ausbildungsmöglichkeiten in den europäischen Ländern wurden anhand deskriptiver statistischer Methoden ausgewertet. Die Analysen wurden mit SPSS V19.0 (SPSS Inc. Chicago, IL) vorgenommen.

Ergebnisse

Experten aus 23 von 35 Ländern nahmen an der Umfrage teil (siehe **Tabelle 1**). Von diesen waren 10 Vorsitzende der entsprechenden nationalen ECP Organisationen. Die Teilnehmer waren vorwiegend weiblich (n = 15; 65,2%) mit einem mittleren Alter von 32,3 Jahren (SD = 3,7). Die Teilnehmer waren entweder psychiatrische Assistenzärzte (n = 11; 47,8%) oder ECP (n = 12; 52,2%). Die Mehrheit der Befragten (n = 17; 73,9%) gaben an, im Vormonat mindestens einen Patienten mit einer PTBS gesehen zu haben, acht Teilnehmer (34,8%) hatten in der Vorwoche einen Patienten gesehen. Nationale Richtlinien für die Behandlung waren in 11 Ländern verfügbar (47,8%). Deren Einfluss wurde nur in vier Ländern (Kroatien, Niederlande, Rumänien, Schweiz) als hoch eingeschätzt. Die Verfügbarkeit der evidenzbasierten Therapien bei PTBS wird in **Tabelle 1** zusammengefasst. Die Angabe „in der Mehrzahl der Ausbildungsinstitute verfügbar“ wurde für theoretische Seminare (n = 10; 43,5%)**,** klinische Fallbesprechungen (n = 9; 39,1%), individuelle Supervision (n = 7; 30,4%), Gruppensupervision (n = 3, 13,0%) und Continuing Medical Education (CME) Kurse (n = 5; 21,7%) genannt. Als Gründe für die unzureichende Implementierung evidenzbasierter Therapien wurden von den Experten (Mehrfachnennungen möglich): fehlende finanzielle Mittel (n = 8; 34,7%), fehlende Expertise (n = 8; 34,7%), unzureichende Anerkennung und Identifizierung von traumaassoziierten Erkrankungen durch Psychiater (n = 3; 13,1%) sowie Probleme bei der Organisation der Versorgung psychisch Kranker (n = 3; 31,1%) angegeben.

Vier Experten berichteten von keinen Problemen mit der Implementierung in ihren Ländern (Finnland, Deutschland, Portugal, Rumänien).

Diskussion

Die von nationalen Experten erhobenen Daten repräsentieren eine Momentaufnahme der aktuellen Bereitstellung evidenzbasierter Therapien bei PTBS und ihrer Präsenz in nationalen Ausbildungscurricula in europäischen Ländern. Unsere Ergebnisse unterstreichen die Notwendigkeit, die Verfügbarkeit evidenzbasierter Therapien der PTBS in europäischen Ländern zu erhöhen. Pharmakologische Behandlungsoptionen bei PTBS waren am häufigsten verfügbar. Da Psychopharmakologie ein integraler Bestandteil der psychiatrischen Ausbildung ist, ist dies wenig verwunderlich. Während Psychoedukation in mehr als 50% der Länder verbreitet war, waren CBT und andere spezifische Behandlungsmöglichkeiten oft nur in spezialisierten Zentren verfügbar. Eine gute Verfügbarkeit für Ausbildungsmöglichkeiten für evidenzbasierte Therapien außerhalb spezialisierter Zentren war, mit Ausnahme von Serbien, vorwiegend in nordeuropäischen Ländern gegeben (Finnland, Deutschland, Niederlande, Malta). Fehlende finanzielle Mittel und fehlende Expertise in den Ländern wurden als die häufigsten Ursachen für die fehlende Implementierung evidenzbasierter Praktiken angegeben. Auch wenn in den nationalen Ausbildungscurricula ein spezifisches Training vorgehalten wurde, fand dies hauptsächlich auf der Basis theoretischer Seminare statt. In nur 30% der Länder wurden CME Programme für die Behandlung von PTBS angeboten. Diese Ergebnisse spiegeln Ergebnisse von Studien wider, die Unterschiede in den Ausbildungscurricula in den europäischen Ländern unterstreichen (Lotz-Rambaldi, Schafer, ten Doesschate, & Hohagen, 2008), eine Situation, die verbessert werden sollte. Theoretische Seminare reichen nicht aus, um eine Verbesserung der Expertise in der klinischen Praxis herbeizuführen. Ausbildungscurricula sollten nicht nur Vorlesungen, sondern auch interaktive Workshops sowie auch ein Vorgehen beinhalten, das die praktische Arbeit überprüft und validiert (Toot, Orrell, Rymaszewska, & Ihl, 2012). Befunde legen nahe, dass Trainingsmaßnahmen die Identifizierung traumassoziierter Erkrankungen verbessern helfen

(Frueh et al., 2002) und psychiatrische Assistenzärzte bei der Ausführung basaler CBT Maßnahmen in Notfällen unterstützen kann (Hamblen, Norris, Gibson, & Lee, 2010). Mögliche Schritte in Richtung einer Implementierung von evidenzbasierten Therapien bei PTBS könnten durch gemeinsame Initiativen auf europäischer Ebene unterstützt werden. Es gibt bereits eine Anzahl Bemühungen, z. B. ein Zertifikat Psychotrauma sowie das European Network for Traumatic Stress (TENTS) der European Society for Traumatic Stress Studies (ESTSS) (Bisson, 2013) und die European Guideline for Target Group-Oriented Psychosocial Aftercare (EUTOPA) (Vymetal et al., 2011). “Summer Schools”, organisiert z. B. durch die EPA (Riese, Pantovic, Fiorillo, Tasman, & Sartorius, im Druck), könnten als Modell für trans-europäische Trainingsinitiativen dienen. Potentielle Sprachbarrieren könnten durch die Implementierung von ähnlichen Maßnahmen auf nationaler Ebene überwunden werden. Internet-basierte Schulungen und Ausbildung von Mentoren wurden ebenfalls als mögliche Strategien vorgeschlagen (Ruzek & Rosen, 2009). Bei der Bereitstellung von finanziellen Ressourcen sollte in Betracht gezogen werden, dass eine qualifizierte Behandlung der PTBS eine Behinderung durch chronische Erkrankung reduzieren kann (Ehlers, Clark, Hackmann, McManus, & Fennell, 2005).

Limitation unserer Studie beinhalten eine nur zufriedenstellende Rücklaufrate (23 von 35) mit einer hohen Prävalenz von Antworten aus osteuropäischen Ländern (14 von 23). Darüber hinaus könnte sich ein Bias daraus ergeben, dass nur ein Experte pro Land geantwortet hat und die Ergebnisse möglicherweise eine subjektive Perspektive darstellen. Trotzdem werden häufig regional bestimmte Experten eingesetzt, um repräsentative Daten zu erheben (Kuzman et al., 2012, Lotz-Rambaldi et al., 2008). Zudem waren die Teilnehmer über die Situation in ihren Ländern gut informiert. Aufgrund der Tatsache, dass nur sehr wenige Daten über Ausbildungsmöglichkeiten in den entsprechenden Ländern verfügbar sind, sollten

Fachgesellschaften für Psychotraumatologie Untersuchungen zu diesem Thema in Europa anstellen.

Strategien um die Verfügbarkeit evidenzbasierter Therapien bei PTBS in europäischen Ländern zu erhöhen sind notwendig. Die Sicherstellung eines höchst möglichen Standards für die Ausbildung der Behandlung von PTBS als Teil der nationalen psychiatrischen Ausbildung ist ein wichtiger Bestandteil dieser Strategien. Das Fehlen ausgebildeter Fachleute kann die Wahrscheinlichkeit senken, PTBS zu erkennen und die Bereitstellung evidenzbasierter Behandlungen auf spezialisierte Zentren begrenzen.

Danksagung

Wir möchten den Mitarbeitern des European Psychiatric Association Büros, speziell Zhanna Zhussupova, danken. Wir möchten uns auch bei den Teilnehmern der Umfrage für ihren wertvollen Beitrag bedanken. Bei der Publikation werden wir von der Deutsche Forschungsgemeinschaft und dem Open-Access-Publikationsfonds der Westfälischen Wilhelms-Universität Münster unterstützt.

Beiträge

D.G., O.A. und N.J. haben die Studie entworfen und die Daten gesammelt. K.K. und D.G. haben die Datenanalyse und Literaturrecherche durchgeführt und die erste Version des Manuskripts verfasst. K.K., D.G., O.A. und N.J. haben das Manuskript kritisch gegengelesen und die aktuelle Version freigegeben.

Literatur

Alonso, J., Angermeyer, M. C., Bernert, S., Bruffaerts, R., Brugha, T. S., Bryson, H., . . . ESEMeD/MHEDEA 2000 Investigators, European Study of the Epidemiology of Mental Disorders (ESEMeD) Project (2004). Use of mental health services in Europe: Results from the European study of the epidemiology of mental disorders (ESEMeD) project. *Acta Psychiatrica Scandinavica.Supplementum, 420,* 47-54. doi:10.1111/j.1600-0047.2004.00330.x

Asukai, N., Tsuruta, N., & Saito, A. (2011). Pilot study on traumatic grief treatment program for Japanese women bereaved by violent death. *Journal of Traumatic Stress, 24*(4), 470-473. doi:10.1002/jts.20662

Bisson, J., & Andrew, M. (2007). Psychological treatment of post-traumatic stress disorder (PTSD). *The Cochrane Database of Systematic Reviews, 3,* CD003388. doi:10.1002/14651858.CD003388.pub3

Bisson, J. (2013). Disseminating and implementing evidence-based practice. *European Journal of Psychotraumatology, 4,* 10.3402/ejpt.v4i0.21252. Print 2013. doi:10.3402/ejpt.v4i0.21252

Bryant, R. A., Ekasawin, S., Chakrabhand, S., Suwanmitri, S., Duangchun, O., & Chantaluckwong, T. (2011). A randomized controlled effectiveness trial of cognitive behavior therapy for post-traumatic stress disorder in terrorist-affected people in Thailand. *World Psychiatry : Official Journal of the World Psychiatric Association (WPA), 10*(3), 205-209. doi:10.1002/j.2051-5545.2011.tb00058.x

Cook, J. M., Rehman, O., Bufka, L., Dinnen, S., & Courtois, C. (2011). Responses of a sample of practicing psychologists to questions about clinical work with trauma and interest in specialized training. *Psychological Trauma : Theory, Research, Practice and Policy, 3*(3), 253-257. doi:10.1037/a0025048

Courtois, C. A. (2001). Traumatic stress studies: The need for curricula inclusion. *Journal of Trauma Practice, 1*, 33-58. doi:10.1300/J189v01n01_03

Courtois, C. A., & Gold, S. N. (2009). The need for inclusion of psychological trauma in the professional curriculum: a call to action. *Psychological Trauma: Theory, Research, Practice, and Policy, 1*(1), 3-23. doi:http://dx.doi.org/10.1037/a0015224

Darves-Bornoz, J. M., Alonso, J., de Girolamo, G., de Graaf, R., Haro, J. M., Kovess-Masfety, V., . . . ESEMeD/MHEDEA 2000 Investigators (2008). Main traumatic events in Europe: PTSD in the European study of the epidemiology of mental disorders survey. *Journal of Traumatic Stress, 21*(5), 455-462. doi:10.1002/jts.20357

Ehlers, A., Clark, D. M., Hackmann, A., McManus, F., & Fennell, M. (2005). Cognitive therapy for post-traumatic stress disorder: Development and evaluation. *Behaviour Research and Therapy, 43*(4), 413-431. doi:S0005-7967(04)00088-9

Frueh, B. C., Cousins, V. C., Hiers, T. G., Cavenaugh, S. D., Cusack, K. J., & Santos, A. B. (2002). The need for trauma assessment and related clinical services in a state-funded mental health system. *Community Mental Health Journal, 38*(4), 351-356. doi:10.1023/A:1015909611028

Hamblen, J. L., Norris, F. H., Gibson, L., & Lee, L. (2010). Training community therapists to deliver cognitive behavioral therapy in the aftermath of disaster. *International Journal of Emergency Mental Health, 12*(1), 33-40. doi:doi.apa.org/?uid=2010-17829-006

Karlin, B.E., Ruzek, J.I., Chard, K.M., Eftekhari, A., Monson, C.M., Hembree, E.A., Resick, P.A., Foa, E.B. (2010), Dissemination of evidence-based psychological treatments for posttraumatic stress disorder in the Veterans Health Administration. *Journal of Traumatic Stress., 23(6), 663-73.* doi: 10.1002/jts.20588

Kuzman, M. R., Giacco, D., Simmons, M., Wuyts, P., Bausch-Becker, N., Favre, G., & Nawka, A. (2012). Psychiatry training in Europe: Views from the trenches. *Medical Teacher, 34*(10), e708-17. doi:10.3109/0142159X.2012.687481

Lotz-Rambaldi, W., Schafer, I., ten Doesschate, R., & Hohagen, F. (2008). Specialist training in psychiatry in Europe-results of the UEMS-survey. *European Psychiatry : The Journal of the Association of European Psychiatrists, 23*(3), 157-168. doi:10.1016/j.eurpsy.2007.12.001

Oflaz, F., Hatipoglu, S., & Aydin, H. (2008). Effectiveness of psychoeducation intervention on post-traumatic stress disorder and coping styles of earthquake survivors. *Journal of Clinical Nursing, 17*(5), 677-687. doi:10.1111/j.1365-2702.2007.02047.x

Ravindran, L. N., & Stein, M. B. (2010). Pharmacotherapy of post-traumatic stress disorder. In M. B. Stein, & T. Steckler (Eds.), *Behavioral neurobiology of anxiety and its treatment* (pp. 505-525). Berlin Heidelberg Deutschland: Springer. doi:10.1007/7854_2009_15

Riese, F., Pantovic, M., Fiorillo, A., Tasman, A., & Sartorius, N. (im Druck). Building an academic career in psychiatry. In Fiorillo, A., Volpe, U., & Bhugra, D. (Eds.), *Psychiatry in practice: A guide for early career psychiatrists*. Oxford University Press.

Roberts, N. P., Kitchiner, N. J., Kenardy, J., & Bisson, J. I. (2010). Early psychological interventions to treat acute traumatic stress symptoms. *The Cochrane Database of Systematic Reviews, 3:CD007944.* doi:10.1002/14651858.CD007944.pub2

Ruzek, J.I.& Rosen, R.C. (2009). Disseminating evidence-based treatments for PTSD in organizational settings: A high priority focus area. *Behaviour Research and Therapy, 47(11), 980-989.* doi: 10.1016/j.brat.2009.07.008

Toot, S., Orrell, M., Rymaszewska, J., & Ihl, R. (2012). A survey of geriatric psychiatry training across Europe. *International Psychogeriatrics / IPA*, *24*(5), 803-808. doi:10.1017/S1041610211002341

Vymetal, S., Deistler, A., Bering, R., Schedlich, C., Rooze, M., Orengo, F., . . . Krtickova, M. (2011). European commission project: European guideline for target group-oriented psychosocial aftercare-implementation. *Prehospital and Disaster Medicine, 26*(3), 234-236. doi:10.1017/S1049023X11006303

Yager, J., Kay, J. (2003). Assessing psychotherapy competence in psychiatric residents: getting real. *Harvard Review of Psychiatry, 11(2),*109-112. doi:10.1176/appi.ap.35.5.283

**Land**	**Evidenzbasierte Therapien**					
	Psychopharmakotherapie	***Psychoedukation***	***CBT***	***EMDR***	***Stress Reduktion***	***Gruppentherapie***
Albanien	Alle	Spezialisiert	Spezialisiert	-	-	-
Aserbaidschan	Mehrheit	Spezialisiert	Selten	Selten	Selten	Selten
Belarus	Alle	Mehrheit	Wenige	Selten	Wenige	Selten
Bosnien und Herzegowina	Alle	Spezialisiert	Spezialisiert	Spezialisiert	Spezialisiert	Spezialisiert
Kroatien	Alle	Mehrheit	Wenige	Spezialisiert	Spezialisiert	Spezialisiert
Tschechische Republik	Alle	Mehrheit	Wenige	Spezialisiert	Wenige	Spezialisiert
Finnland	Alle	Mehrheit	Mehrheit	Wenige	Alle	Wenige
Frankreich	Mehrheit	Wenige	Mehrheit	Spezialisiert	Wenige	Spezialisiert
Deutschland	Alle	Mehrheit	Spezialisiert	Spezialisiert	Alle	Wenige
Griechenland	Wenige	Spezialisiert	Spezialisiert	Selten	Wenige	Spezialisiert
Lettland	Alle	Wenige	Selten	Selten	Selten	Selten
Litauen	Alle	Wenige	Wenige	Selten	Selten	Selten
Malta	Alle	Alle	Selten	Selten	Mehrheit	Selten
Niederlande	Alle	Alle	Alle	Mehrheit	Alle	Wenige
Polen	Wenige	Mehrheit	Wenige	Selten	Wenige	Selten

Portugal	Alle	Wenige	Mehrheit	Selten	Wenige	Wenige
Rumänien	Mehrheit	Mehrheit	Wenige	Selten	Wenige	Selten
Russland	Alle	Spezialisiert	Spezialisiert	Selten	Spezialisiert	Spezialisiert
Serbien	Alle	Alle	Mehrheit	Wenige	Alle	Spezialisiert
Slowenien	Alle	Alle	Wenige	Selten	Wenige	Spezialisiert
Schweiz	Alle	Alle	Alle	Wenige	Alle	Wenige
Türkei	Wenige	Wenige	Wenige	Selten	Wenige	Spezialisiert
Ukraine	Alle	Selten	Wenige	Selten	Wenige	Wenige

Tabelle1: Verfügbarkeit evidenzbasierter Therapien bei PTBS und Ausbildungsmöglichkeiten in den teilnehmenden Ländern (“Alle” = in fast allen Zentren; “Mehrheit” = in der Mehrheit der Zentren; “Wenige” = in wenigen Zentren; “Spezialisiert” = nur in spezialisierten Zentren; “Selten” = sehr selten verfügbar; “-“ = keine Behandlung/Ausbildung erhältlich).
